# Stock Market Exposure and Anxiety in a Turbulent Market: Evidence From China

**DOI:** 10.3389/fpsyg.2019.00328

**Published:** 2019-02-19

**Authors:** Xin Qin, Hui Liao, Xiaoming Zheng, Xin Liu

**Affiliations:** ^1^Sun Yat-sen Business School, Sun Yat-sen University, Guangzhou, China; ^2^Robert H. Smith School of Business, University of Maryland, Rockville, MD, United States; ^3^School of Economics and Management, Tsinghua University, Beijing, China; ^4^Renmin Business School, Renmin University of China, Beijing, China

**Keywords:** stock, anxiety disorder, mental health, health psychology, big data

## Abstract

The stock market in China has experienced significant turbulence since July 2014, including a bull market. In this paper, we propose that exposure to stock (defined as the condition of being exposed to both stock and stock-related information) can induce anxiety disorder when the market is in a turbulent period. To examine this prediction, we designed two studies in which we operationalized exposure to stock in two different ways. In Study 1, a panel analysis of a longitudinal data set for the Chinese stock market from January 2014 to July 2015 demonstrated that exposure to stock had a significant positive impact on individuals’ anxiety disorder, even in a bull market. Study 2, employing priming experiments, further supported that a temporarily primed “stock mindset” subconsciously increased participants’ anxiety. In addition, Study 2 revealed that physical exercise helped attenuate the detrimental impact of exposure to stock on mental well-being. This research demonstrates the detrimental impact of exposure to a turbulent stock market – even a rising market – on individuals’ mental health. Furthermore, it identifies an effective way to buffer such impact, and suggests ways for social scientists to employ search engines and the related data sets to obtain psychological or behavioral information (especially emotions and emotion disorders) by examining longitudinal “Big Data.”

## Introduction

The stock market in China has received increasing attention since July 2014. The Shanghai Stock Exchange (SSE) Composite Index, a key indicator of the Chinese stock market provided by the SSE, has fluctuated dramatically. From January to July of 2014, the Index declined from 2109.387 to 2050.381, but by December 2014 had risen to 3234.677. The peak occurred on June 12, 2015, when the Index hit 5166.350. By July 31, 2015, however, the average had plummeted to 3663.726. Meanwhile, the number of accounts for all listed stocks (A shares) in both the SSE and the Shenzhen Stock Exchange (SZSE) rose from 181,403,520 in January 2014, to 235,975,259 in June 2015 – that is, more than one-fifth of Chinese adults speculated in the stock market. Notably, 12,894,626 new accounts were added in April 2015 alone^1^. Given such a wide range of potential influence, exposure to stock conceptualized as the condition of being exposed to both stock and stock-related information is a key issue that warrants further investigation in current Chinese society.

Looking into the literature, most extant economic theories and findings have assumed that decreases in stock markets have a negative effect on mental health (Gili et al., 2013; McInerney et al., 2013; Schwandt, 2014; Cotti et al., 2015; Lin et al., 2015; Engelberg and Parsons, 2016), whereas increases lead to improvements in mental health and life satisfaction (Frijters et al., 2015). Similarly, some other studies have found that fluctuations in housing wealth have critical psychological impacts (Fichera and Gathergood, 2016; Heiss et al., 2016; Erixson, 2017). These findings seem to support the “money buys happiness” argument (Stevenson and Wolfers, 2013).

However, with the rise and fluctuations of the stock market in China since July 2014, a number of journalists have documented the negative consequences of the stock market’s turbulence on stock investors – and even on persons who do not invest in stocks. For instance, a psychiatrist in Wenzhou noted, “In the past 2 weeks, three to six persons every day have been coming to receive psychological treatment for stock-related issues,” with most of them suffering from such concerns as insomnia, anxiety, and depression. As an example, this doctor cited a patient who would shake and feel chest tightness whenever the word “stock” or anything related to stocks was mentioned – whether by another person or on television^2^. Taken together, examining the psychological consequences of exposure to stock in China, a turbulent stock market, might reveal findings different from those provided by the extant research.

Accordingly, our research examines the psychological consequences of exposure to stock in a turbulent stock market. In particular, we focus on anxiety disorder rather than other types of psychological disorders, because generalized anxiety disorder (GAD) is one of the most common psychological disorders, prevalent in both general medical practice (approximately 2.8 to 8.5%) (Leon et al., 1995; Olfson et al., 1997; Roy-Byrne and Wagner, 2004) and in the general population (approximately 1.6 to 5.0%) (Wittchen et al., 1994; Kessler et al., 2001, 2005). The American Psychiatric Association’s *Diagnostic and Statistical Manual of Mental Disorders, Fifth Edition* (*DSM-5*) defines anxiety as a mental disorder characterized by excessive, uncontrollable, and often irrational worry – that is, apprehensive expectation about events or activities; the physical symptoms of anxiety include fidgeting, headaches, difficulty concentrating, trembling, irritability, sweating, restlessness, and insomnia (American Psychiatric Association, 2013). Anxiety disorder is particularly detrimental to individuals’ health, with its development increasing the occurrence of disability in the workplace (Ballenger et al., 2001; Zayfert et al., 2002; Comer et al., 2011). Given the widespread nature of this disorder and its effects on all facets of life, the anxiety disorder arising in persons exposed to the stock market as a consequence of the recent turbulence in the Chinese stock market represents an important phenomenon, and one that is the subject of the present research.

Departing from prior theories and findings that focus on the negative effects that a stock market crash imposes on mental health, our research suggests that in a turbulent stock market, exposure to stock (defined as the condition of being exposed to both stock and stock-related issues) may trigger heightened anxiety even when the market is rising (i.e., a bull market). Specifically, financial security – the condition of maintaining stability of income or other financial resources for the present time and for the foreseeable future – is related to a fundamental human need for safety (Maslow, 1943). Although a turbulent stock market is associated with opportunities, it violates people’s sense of financial security, as income or return expectations are in flux. The actual losses and potential losses of a fluctuating market create uncertainty about financial security, which in turn induces anxiety. That is, in a turbulent stock market, consideration of gain impacts investors in the same way that they are affected by consideration of loss. As numerous stock commentators have stated, “Buying stocks is like playing the heartbeat,” and “Heart rate fluctuates according to the stock market prices” (www.cctv.com, 2007). These arguments are also in line with theories of loss aversion (including prospect theory), which propose that people are more sensitive to losses than to gains due to the inherent tendency of preferring avoiding losses to obtaining gains, and they fight very hard to keep what they currently possess (Kahneman and Tversky, 1979; Tversky and Kahneman, 1991). Even in a bull market, investors may be concerned about the possibility of losing their current assets. As a result, many investors experience pressure to maintain, or to exceed, their performance levels, which in turn leads them to feel anxious.

To examine our expectations of the relationship between exposure to stock and prevalence of anxiety, we analyzed longitudinal data from the Chinese stock market between January 2014 and July 2015 (Study 1), and we conducted priming experiments (Study 2) in which we operationalized exposure to stock in two different ways. In the panel analysis conducted in Study 1, we found that exposure to stock had a strong impact on individuals’ psychological state (i.e., anxiety disorder). During the January 2014 to July 2015 period, when even more Chinese individuals experienced exposure to stock (as measured by the *Baidu Index*), the prevalence of anxiety disorder among individuals with such exposure increased 437%. The results of Study 2 further demonstrate that, on a cognitive level, a temporarily primed “stock mindset” increased participant anxiety. These studies complement each other and together provide evidence for the causal impact of exposure to stock in a turbulent stock market on anxiety disorder, with the results having high external and internal validity.

This paper makes several unique contributions that enhance understanding of the psychological consequences of exposure to stock in a turbulent stock market. First, this research reveals that exposure to stock induces anxiety, no matter whether stock prices go up or go down. The prior literature has emphasized the detrimental effects of a falling stock market (Gili et al., 2013; McInerney et al., 2013; Schwandt, 2014; Cotti et al., 2015; Lin et al., 2015; Engelberg and Parsons, 2016). For instance, using data on hospitalization for mental disorders in Taiwan, for a 4000-day period beginning in 1998 and ending in 2009, Lin et al. found that a 1000-point fall in the TAIEX (Taiwan Stock Exchange Capitalization Weighted Stock Index) increased the number of daily hospitalizations for mental disorders by 4.71% (Lin et al., 2015). Similarly, using Australian survey data for 2001–2012, Frijters et al. (2015) found that stock market increases led to a significant but modest improvement in life satisfaction and mental health. Adding to this limited literature, our research is one of the first attempts to demonstrate that, in a turbulent stock market subject to large fluctuations, exposure to stock triggers anxiety disorder, regardless of the general trend of the prices change (up or down).

Second, this research demonstrates an effective approach to measuring anxiety disorder in a daily or weekly manner – namely, through certain key words identified in the *Baidu Index*. In the extant psychological research, scholars have primarily used psychometric scales to measure anxiety disorder (e.g., Spitzer et al., 2006), which is applicable when surveying a small number of participants but hard to reflect this phenomenon on the population level. In comparison, using data from *Baidu Index* can depict people’s overall levels of anxiety disorder within a region or country in a daily or weekly basis. Relatedly, this novel approach introduces a new method of studying the antecedents and consequences of anxiety disorder using panel designs, especially in developing countries where systematic longitudinal (mental) health data systems are lacking. For instance, our approach will be beneficial in future research that explores the impact of anxiety disorder on regional productivity rates, injuries, innovation, and so on. Broadly speaking, our research design encourages future researchers to employ search engines and the related data sets to obtain relevant “Big Data” (especially emotions and emotion disorders) for examining their interested research questions.

## Study 1: Panel Analyses of the Stock Market in China

### Methods

**FIGURE 1:**
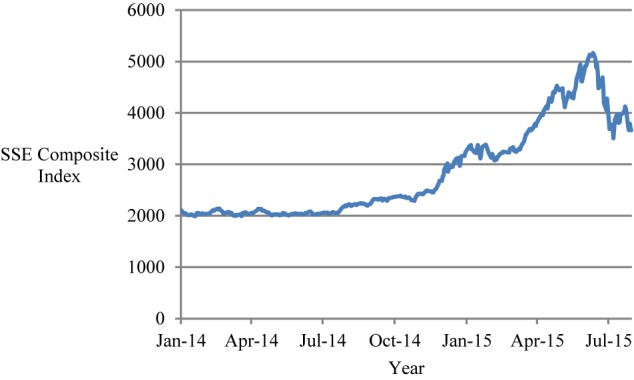
The SSE Composite Index from January 2014 to July 2015.

We collected data for the panel analyses in Study 1 from several sources. First, the weekly *average level of the SSE Composite Index* (for which the natural logarithm was used to reduce heteroscedasticity), its *amplitude* [i.e. (the highest price – the lowest price) / the previous day’s closing price; unit: percent], and *change amount* [i.e. (the closing price – the previous day’s closing price)/the previous day’s closing price; unit: percent], were collected through the *Wind* Datafeed Service, looking at data from January 1, 2014 to July 31, 2015 (for a total of 83 weeks; see Figure 1 and Supplementary Figures S1, S2). The average level, amplitude, and change amount of SSE Composite Index acted as control variables in measuring stock market characteristics.

**FIGURE 2:**
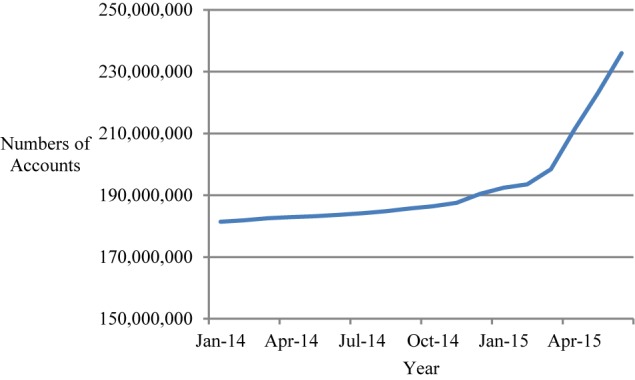
Number of accounts for all listed stocks (A shares) in the SSE and the SZSE from January 2014 to June 2015.

Second, we employed the ratio of the total number of accounts for all listed stocks (A shares) in the SSE and the SZSE divided by the population in the corresponding province (unit: millions) as a proxy to operationalize *exposure to stock*. The main reason for this operationalization is that, when a higher portion of individuals in a province have stock accounts, more people in that province will be exposed to stock and stock-related information directly or indirectly. For example, they are more likely to receive and pay attention to stock-related information, and are more likely to discuss and share such information in their social interactions, which then exposes more people in their families, at work, or through their extended social network to the stock market. The account data were derived from the China Securities Depository and Clearing Corporation Limited (CSDC) data set (see text footnote 1). The CSDC data set records monthly accounts for a set of 31 provinces in China. The account data were collected from January 2014 to June 2015 (for a total of 18 months; see Figure 2 and Supplementary Figures S3, S4). The CSDC data set did not include the province-level account data after June 2015. Therefore, the data for exposure to stock spanned from January 2014 to June 2015, or almost the full cycle of a turbulent stock market period. Thus, the period covered by the data set was long enough to sufficiently represent the turbulent stock market in China. We used population data from the National Bureau of Statistics of China at the end of 2013 to represent the 2014 population, and we used population data at the end of 2014 to represent the 2015 population^3^. We believe that our analyses are not biased by these choices, as the population in each province is relatively stable, especially within a range of 1 or 2 years.

Third, we measured anxiety disorder based on information obtained via the *Baidu Index*, which is similar to *Google Trends* (Baidu is the biggest Chinese search engine in the world, similar to Google in the United States^4^). In our search, we used the keyword “
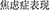
” (*jiaolvzheng biaoxian*, which means “manifestation of anxiety disorder”) and sought relevant data for each of China’s 31 provinces. When collecting data, we complied with the terms of service for the website. Results were divided by individual provincial population (unit: millions), to give the Internet search frequency of this keyword for each province.

We chose the *Baidu Index* approach to measure anxiety disorder for several reasons. First, the Chinese people are gradually becoming accustomed to searching for solutions and treatments for their health problems via the Internet. As a result, the use of health portal websites (i.e., platforms to deliver specific health information and disseminate new health concepts) has recently increased rapidly. For instance, a leading health portal in China found at www.xywy.com (*xunyi wenyao wang*, 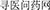; Alexa weekly rank: 233; June 6, 2015) had more than 80 million registered users, 20 million daily unique visitors, and 300 million monthly unique visitors by the end of 2014. When people consult or search for health information, they often start with Baidu.com, as it is the dominant and most frequently used search engine in China (see StatCounter Global Stats for detailed information, see text footnote 4). Second, compared with physical illnesses, mental illnesses (including anxiety disorder) are more likely to be deemed chronic and non-urgent. Thus, individuals may have a tendency to assume that they can deal with these mental health issues by themselves, by researching mental health information online instead of seeking professional help at clinics. Third, in contrast to their response to physical illnesses, many people in China do not want to talk about mental disorders and are reluctant to receive psychological treatments. They often do not regard mental disorders as true illnesses, or they may perceive mental disorders as shameful conditions. Many Chinese are concerned that they would “lose face” if people found out that they have mental disorders (Chen, 2005; San, 2006), which further increases their desire to search for information relevant to mental health privately, via the Internet.

**FIGURE 3:**
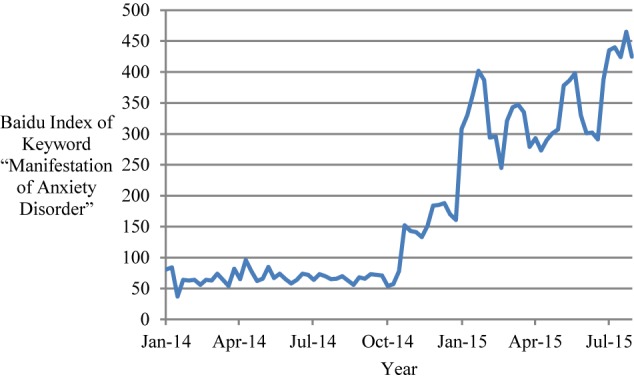
The Baidu Index search results for the keyword “manifestation of anxiety disorder” from January 2014 to July 2015 in China.

These conditions create a logical rationale for employing the *Baidu Index* of “manifestation of anxiety disorder” per capita for each province as a means for assessing the prevalence of anxiety disorder in each province. Notably, individuals who suspect that they have anxiety disorder usually search for the manifestations or symptoms of anxiety disorder, so that they can compare their own experiences with the typical symptoms of anxiety disorder. Therefore, we chose to employ the keyword “manifestation of anxiety disorder” rather than other related keywords (e.g., “anxiety disorder”). The weekly total *Baidu Index* of the “manifestation of anxiety disorder” search for the 31 provinces was collected for the period from January 1, 2014 to July 31, 2015 (for a total of 83 weeks; Figure 3).

Furthermore, to examine the nomological network of the anxiety disorder measure, we consider how GDP per capita relates to anxiety disorder. Based on the generalization that people in developed regions tend to have a faster pace of life and a higher level of life pressure (e.g., due to high costs of housing or to the pursuit of professional advancement), it is logical to expect that economic development would be positively correlated with anxiety disorder in China (i.e., an emerging/developing country) (Baxter et al., 2013). As we expected, our analysis revealed that GDP per capita in 2013 was positively associated with anxiety disorder (*r* = 0.432, *p* < 0.001), providing evidence that supports our measure’s criterion-related validity.

### Results and Discussion

We employed fixed effects models for the panel data, which accounted for unmeasured, province-specific effects that differed across provinces but did not change over time (Wooldrige, 2002), to examine the effects of exposure to stock in the turbulent stock market on anxiety disorder (from January 2014 to July 2015, for a total of 82 weeks, for all models except exposure to stock; from January 2014 to June 2015, for a total of 77 weeks, for models with exposure to stock). In addition, we clustered observations at the province level to account for the potential non-independence of observations within each province. Furthermore, we estimated robust errors to account for potential error heteroskedasticity. The summarized statistics for the key variables are presented in Supplementary Table S1. As shown in Table 1, some stock market attributes clearly influenced the prevalence of anxiety disorder, such as the SSE Composite Index (β = 1.427, *p* < 0.001, Model 4) and the amplitude of the SSE Composite Index (β = 0.122, *p* < 0.001, Model 4). Moreover, the results of Model 6 revealed that, after controlling for the stock market’s attributes, exposure to stock positively predicted development of anxiety disorder (β = 11.531, *p* < 0.01), confirming our expectation. Also, further analyses revealed that anxiety disorder did not significantly predict exposure to stock (β = 0.005, *ns*), indicating there was no reciprocal relationship between exposure to stock and anxiety disorder.

**Table 1:** Impact of exposure to stock on anxiety disorder (panel analyses).

Variable	Model 1	Model 2	Model 3	Model 4	Model 5	Model 6
SSE Composite Index	1.822^∗∗∗^			1.427^∗∗∗^		1.185^∗∗∗^
(log)	(7.851)			(6.738)		(7.769)
Amplitude of SSE		0.317^∗∗∗^		0.122^∗∗∗^		0.032^∗^
Composite Index		(8.659)		(7.605)		(2.322)
Change amount of SSE			-0.018	0.014		0.033^∗^
Composite Index			(-1.621)	(1.082)		(2.056)
Exposure to stock					26.094^∗∗∗^	11.531^∗∗^
					(5.625)	(3.433)
Constant	-13.733^∗∗∗^	0.044	0.681^∗∗∗^	-10.853^∗∗∗^	-3.312^∗∗∗^	-10.542^∗∗∗^
	(-7.481)	(0.606)	(367.518)	(-6.443)	(-4.764)	(-8.686)
*R*2	0.414	0.293	0.0003	0.437	0.290	0.442
*F*	61.633^∗∗∗^	74.980^∗∗∗^	2.629	29.899^∗∗∗^	31.637^∗∗∗^	25.801^∗∗∗^


*n = 2542, from January 2014 to July 2015, total 82 weeks, for all models except exposure to stock; n = 2387, from January 2014 to June 2015, total 77 weeks, for models with exposure to stock. t statistics in parentheses. ^∗^Significant at 5%, ^∗∗^significant at 1%, ^∗∗∗^significant at 0.1%.*

Furthermore, when we used a different way to assess the dependent variable, we obtained the same pattern of results. Specifically, instead of using the search frequency of the term “manifestation of anxiety disorder,” we used the search frequency of the term “anxiety disorder,” as the dependent variable. We found that the search frequencies of “manifestation of anxiety disorder” and “anxiety disorder” were positively correlated with each other (*r* = 0.432, *p* < 0.001). In addition, we found that exposure to stock also significantly predicted the search frequency of the “anxiety disorder” keyword (β = 11.480, *p* < 0.01; see Supplementary Table S2). These data suggest that our results were robust and consistent when using two different measures of anxiety disorder.

Finally, we tested our predictions using the bull market data only (from January 1, 2014 to June 12, 2015, for a total of 75 weeks; see Supplementary Tables S3, S4). The results indicated that exposure to stock over this period positively predicted anxiety disorder (β = 13.232, *p* < 0.01). Thus, even during the economic upswings associated with bull markets, exposure to stock induces anxiety disorder.

## Study 2: Priming Experiments

### Methods

#### Participants

To establish the causality of exposure to stock in a volatile stock market in leading to higher prevalence of anxiety disorder, we conducted an experiment in which we manipulated exposure to stock.

Study 2 was carried out in accordance with the recommendations of the research ethics committee in a large university in China. The protocol was approved by the research ethics committee in this university. All subjects gave written informed consent in accordance with the Declaration of Helsinki. Prior to beginning the study, we obtained behavioral consent from all participants; all the participants chose “I voluntarily participate this experiment” in a multiple-choice question. All the research materials were written in Chinese. In addition, all participants were well informed that they were free to quit the experiment at any time.

To systematically evaluate the priming effects of stock, we conducted the experiments twice in the context of the Chinese stock market’s fall and rise (Study 2a and Study 2b, respectively), during the period of July 2, 2015 to July 21, 2015, when the overall stock market was turbulent. Specifically, we conducted the priming experiments when the stock market declined (July 8, 2015; the average change amount of the SSE Composite Index from the preceding week = –2.806%; Study 2a) and increased (July 21, 2015; the average change amount of the SSE Composite Index from the preceding week = 0.492%; Study 2b). In Study 2a, the change amounts for July 2 and 3, and for July 6, 7, and 8, 2015, were (respectively) -3.477, -5.772, 2.414, -1.292, and –5.901%. In Study 2b, the change amounts for July 15, 16, and 17, and for July 20 and 21, 2015, were (respectively) -3.027, 0.459, 3.510, 0.878, and 0.640%. Study 2a included 153 people from a cross-national Chinese sample as participants, while 221 people participated in Study 2b. The sample sizes were determined in advance based on a power analysis so that there was at least a 95% chance of detecting a small-medium effect size (Faul et al., 2007). Among participants of Study 2a, 59.5% were female, the average age was 32.5 years, and the average education was 15.8 years. Among participants of Study 2b, 50.7% were female, the average age was 31.9 years, and the average education was 15.7 years. For completing the two-part study online, participants received the opportunity to obtain a gift that cost $5.

#### Procedures

In the first part of the study, participants were told that this experiment explored individual differences of linguistic competence. Participants were randomly assigned to one of two priming conditions: *stock mindset* versus *control*. In line with the prior psychological literature, we chose the commonly used scrambled sentence test to activate participants’ momentary mindset (Chartrand and Bargh, 1996; Bargh and Chartrand, 1999). Participants were asked to use a set of five randomly positioned words and phrases to construct grammatically correct four-word sentences. In the stock-mindset condition, the five groups of words included words related to stocks [e.g., “stock” (*gupiao*, 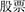), “stock market” (*gushi*, 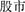), and “investing in stocks” (*chaogu*, 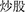)]. The control condition included no words related to stocks. In Study 2a, five sentences were provided to both the stock priming and the control groups. Study 2b used the same procedure except that there were seven sentences, two of which were added as filler sentences (the same for two conditions). Participants constructed four-word sentences from a set of five randomly positioned words, including those related to stocks (e.g., “stock”), in the stock priming condition; they did the same sentence task with words unrelated to stocks (e.g., “education”) in the control condition.

A 1-min filler task (i.e., filling in their demographic information) was selected to mask the purpose of the study. Participants were then instructed to participate in an ostensibly independent study, in which the researchers were interested into their lives and work status. Then, in the context of an ostensibly independent study, participants were asked to report their level of anxiety via a commonly used seven-item GAD scale (α = 0.95 in Study 2a; α = 0.93 in Study 2b) (Spitzer et al., 2006). This scale has been cited 1562 times since it was published (July 10, 2015). Finally, participants responded to post-experiment funnel debriefing questions about their awareness of the purposes of the study and the experimental manipulation (e.g., “What was the purpose of this study?”) (Bargh and Chartrand, 2000).

### Results and Discussion

In line with our prediction, participants reported higher levels of anxiety when they were primed to activate a stock mindset (Study 2a: *M* = 2.716, *SD* = 0.939, when stock market went down; Study 2b: *M* = 2.500, *SD* = 0.939, when stock market went up) than did the participants in the control condition [Study 2a: *M* = 2.199, *SD* = 0.965; *t*(151) = 3.362, *p* < 0.001, Cohen’s *d* = 0.544, η^2^ = 0.070, when stock market went down; Study 2b: *M* = 2.097, *SD* = 0.749; *t*(219) = 3.539, *p* < 0.001, Cohen’s *d* = 0.476, η^2^ = 0.054, when stock market went up].

Additional analyses provided further insights into the impact of exposure to stock on anxiety, as well as ways to buffer this impact. In Study 2b, we included measures of whether the participants or their family members bought stocks (yes = 1, no = 0) and of participants’ weekly exercise frequency (unit: times). The results revealed that participants who bought stocks, or whose family members bought stocks, experienced higher rates of anxiety disorder (*M* = 2.350, *SD* = 0.936) than those who did not (*M* = 2.186, *SD* = 0.711), but not significantly [*t*(219) = 1.325, *p* = 0.187]. Meanwhile, the exposure to stock × buying stocks interaction term did not significantly predict anxiety disorder (β = 0.103, *p* = 0.672). The results suggest that exposure to stock during a turbulent stock market triggered anxiety in a universal manner, no matter whether or not participants or their family members actually bought stocks.

Further analysis revealed that weekly exercise frequency interacted with exposure to stock to influence the prevalence of anxiety disorder (β = –0.143, *p* < 0.05). Simple slope analysis indicated that the effect of exposure to stock on anxiety disorder prevalence was significantly positive when weekly exercise frequency was low (β = 0.683, *t* = 4.448, *p* < 0.001), but the effect was positive though not significant when weekly exercise frequency was high (β = 0.121, *t* = 0.790, *p* = 0.430) (Supplementary Figure S5). In particular, further analyses indicated that exercising 4.404 times per week was able to totally buffer the effect of exposure to stock (β = 0.246, *t* = 1.970, *p* = 0.050). This result suggests that exercise may be a valuable way to attenuate the detrimental impact of exposure to stock on mental well-being.

In Study 2a, the funnel debriefing revealed that no participant identified the purpose of the experiment and no one thought the sentence task influenced their responses later. In Study 2b, three out of the 224 participants suggested that the purpose of the experiment was to explore how buying stocks affected individuals’ psychology or suggested that “stock” appeared multiple times, so we excluded the data for those three individuals from subsequent analyses (Bargh and Chartrand, 2000). Overall, these results demonstrated that, even when activated temporarily, in the condition of a turbulent stock market a stock mindset subconsciously induced anxiety.

## Discussion

Our findings demonstrate that exposure to stock in a turbulent stock market has a significant negative impact on individuals’ mental health. The turbulence serves as a prerequisite of these findings. More specifically, panel analyses of the highly volatile stock market in China from January 2014 to July 2015 revealed that exposure to stock was positively associated with individuals’ anxiety disorder, even though the market generally went up during that period of time. A controlled priming experiment further supported our expectation that exposure to stock in a turbulent stock market triggered anxiety without awareness, which helped establish causality.

Our findings reveal that exposure to stock induces anxiety in a turbulent stock market – even in a bull market. These findings have important practical implications. First, it is important to note that the results do not mean that growth of the stock market should be discouraged or the stock market should be maintained at a constant level. However, when vigorously developing financial markets, the government does need to recognize the potentially detrimental effects of exposure to a turbulent or growing stock market on the psychological well-being of the public. In addition, while this research illustrated the significant impact that exposure to stock in a turbulent stock market can impose on individuals in the area of anxiety disorder and deepened our understanding of the antecedents of anxiety disorder, more research is needed to explore the impacts on other types of mental disorders, as well as on stress-related physical illnesses such as hypertension and cardiovascular diseases. More broadly, because cognitive distraction undermines job engagement and productivity (Lee et al., 2014), it is worth investigating how exposure to stock influences employees’ workplace behaviors and effectiveness. On the basis of ego depletion theory or conservation of resources theory, we would expect exposure to a turbulent stock market to decrease employees’/leaders’ productivity, but increase their counterproductive/abusive behaviors and workplace injuries (Baumeister et al., 1998; Ju et al., 2016; Qin et al., 2018). Also, it is promising to investigate how the ways of getting exposed to stock-relevant information and the contents of information influence the impacts of exposure to stock on anxiety disorder. For example, compared to exposure to stock via we-media, exposure to stock via official television may have larger impacts on anxiety disorder, as its information is perceived as more trustworthy. Thus, more research is needed to empirically and systematically examine the potential costs of the turbulent stock market and their boundary conditions, even when it is overall a bull market. Research along these lines will contribute to a better understanding of how to alleviate the negative effects of the stock market while enjoying its beneficial aspects.

In recent years, the Chinese government has advocated *the Chinese Dream* (*zhongguo meng*, 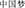); “the Chinese Dream” is a term popularized after 2013, representing a series of personal and national ideals of the “Chinese nation,” which primarily emphasize the people’s happiness and well-being. Our results suggest that the government – as part of this advocacy – may want to reduce the exposure to stock that people experience in a turbulent stock market, or if such exposure is inevitable, to help people better cope with the anxiety induced by stock market. In addition, individuals who want to minimize anxiety disorder need to reduce their own exposure to stock – e.g., they should read less about stocks in newspaper and magazines, and should limit sharing of information and discussion about stocks in social media. Furthermore, it is necessary to determine which factors have the potential to attenuate the detrimental impact of exposure to stock. In our Study 2b, we found that physical exercise can attenuate the triggering effect of exposure to stock on anxiety disorder. This is consistent with the prior literature which found that physical exercise can serve as a stress-management treatment leading to decrease in negative affect and increase in positive affect (Petruzzello et al., 1991; Long and Stavel, 1995; Raedeke, 2007). Future research is needed to discover other methods or factors (e.g., individual risk-relevant or anxiety-relevant differences) which attenuate the negative effects of exposure to a volatile stock market. Relatedly, although all of our samples were from China, our theoretical arguments are not culturally specific. Thus, we suggest our findings hold in other cultures and countries. Nevertheless, we welcome future research to replicate our findings across cultures.

Another area addressed by our study that deserves further development is the application of search engine indexes for research. The resource known as “Big Data” has attracted an increasing amount of attention in the social sciences recently (McAfee and Brynjolfsson, 2012). An obstacle faced by social scientists is locating Big Data that contain psychological and behavioral variables, which are typically lacking in secondary data. Our research demonstrates a valuable method for obtaining comprehensive data through search engine indexes. In our study, we used Baidu.com to obtain regional data on the number of potential occurrences of anxiety disorder, tracked longitudinally. In future studies, we recommend social scientists to collaborate with the search engine companies so that they can obtain access to even more search results using many other keywords (e.g., “panic attack,” “feeling anxious”). In addition, future research might consider how search engine indexes can be used to examine other important research questions. For instance, through the use of appropriate keyword searches and search engine indexes (e.g., *Google Trends*, *Baidu Index*), we could capture data identifying the level of attention that people pay to the amount of environmental haze (*wumai*, 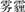) in each region daily, weekly, or monthly.

In summary, the findings presented in this research demonstrate that exposure to stock in a turbulent stock market significantly increases the level of anxiety disorder. To our best knowledge, this is the first study to empirically reveal that, even in an overall bull market, greater prevalence of anxiety disorder is induced by increased exposure to stock. These findings can help both government and the public recognize the increased anxiety associated with stock market volatility, and they highlight the importance of discovering ways to buffer the potential for increased anxiety disorder when the nation’s stock market is turbulent, even when it is growing overall. Moreover, through our creative measurement of regional anxiety disorder in China, we identify an effective approach for social scientists to obtain longitudinal Big Data relevant to human psychology or behavior.

## Author Contributions

XQ, XZ, and HL designed and performed the research. XQ and XL analyzed the data. XQ, XZ, HL, and XL wrote the manuscript.

## Conflict of Interest Statement

The authors declare that the research was conducted in the absence of any commercial or financial relationships that could be construed as a potential conflict of interest.

## Footnotes

1 http://www.chinaclear.cn

2 http://news.xinhuanet.com/legal/2015-07/02/c_127977235.htm

3 http://www.stats.gov.cn

4 http://gs.statcounter.com

## References

[B1] American Psychiatric Association. (2013). *Diagnostic and Statistical Manual of Mental Disorders: DSM-5*, 5th Edn Washington, DC: American Psychiatric Association 10.1176/appi.books.9780890425596

[B2] Ballenger J. C. Davidson J. R. T. Lecrubier Y. Nutt D. J. Borkovec T. D. Rickels K. (2001). Consensus statement on generalized anxiety disorder from the international consensus group on depression and anxiety. *J. Clin. Psychiatry* 62 53–58. 11414552

[B3] Bargh J. A. Chartrand T. L. (1999). The unbearable automaticity of being. *Am. Psychol.* 54 462–479. 10.1037/0003-066X.54.7.462

[B4] Bargh J. A. Chartrand T. L. (2000). “The mind in the middle: a practical guide to priming and automaticity research,” in *Handbook of Research Methods in Social and Personality Psychology*. New York, NY: Cambridge University Press, 253–285.

[B5] Baumeister R. F. Bratslavsky E. Muraven M. Tice D. M. (1998). Ego depletion: is the active self a limited resource? *J. Personal. Soc. Psychol.* 74 1252–1265. 10.1037/0022-3514.74.5.1252 9599441

[B6] Baxter A. J. Scott K. M. Vos T. Whiteford H. A. (2013). Global prevalence of anxiety disorders: a systematic review and meta-regression. *Psychol. Med.* 43 897–910. 10.1017/S003329171200147X 22781489

[B7] Chartrand T. L. Bargh J. A. (1996). Automatic activation of social information processing goals: nonconscious priming reproduces effects of explicit conscious instructions. *J. Personal. Soc. Psychol.* 71 464–478. 10.1037/0022-3514.71.3.464

[B8] Chen W. Q. (2005). Why is it difficult for Chinese people to accept psychological counseling? [in Chinese]. *Beijing Sci. Tech. Rep.* 19 1–2.

[B9] Comer J. S. Blanco C. Hasin D. S. Liu S.-M. Grant B. F. Turner J. B. (2011). Health-related quality of life across the anxiety disorders: results from the National Epidemiologic Survey on Alcohol and Related Conditions (NESARC). *J. Clin. Psychiatry* 72 43–50. 10.4088/JCP.09m05094blu 20816036 PMC3000882

[B10] Cotti C. Dunn R. A. Tefft N. (2015). The dow is killing me: risky health behaviors and the stock market. *Health Econ.* 24 803–821. 10.1002/hec.3062 24803424

[B11] Engelberg J. Parsons C. A. (2016). Worrying about the stock market: evidence from hospital admissions. *J. Finance.* 71 1227–1250. 10.1111/jofi.12386

[B12] Erixson O. (2017). Health responses to a wealth shock: evidence from a swedish tax reform. *J. Popul. Econ.* 30 1281–1336. 10.1007/s00148-017-0651-2

[B13] Faul F. Erdfelder E. Lang A. G. Buchner A. (2007). G^∗^ Power 3: a flexible statistical power analysis program for the social, behavioral, and biomedical sciences. *Behav. Res. Methods* 39 175–191. 10.3758/BF03193146 17695343

[B14] Fichera E. Gathergood J. (2016). Do wealth shocks affect health? New evidence from the housing boom. *Health Econ.* 2 57–69. 10.1002/hec.3431 27870303 PMC5111776

[B15] Frijters P. Johnston D. W. Shields M. A. Sinha K. (2015). A lifecycle perspective of stock market performance and wellbeing. *J. Econ. Behav. Organ.* 112 237–250. 10.1016/j.jebo.2015.02.004

[B16] Gili M. Roca M. Basu S. McKee M. Stuckler D. (2013). The mental health risks of economic crisis in Spain: evidence from primary care centres, 2006 and 2010. *Eur. J. Public Health* 23 103–108. 10.1093/eurpub/cks035 23132877

[B17] Heiss F. McFadden D. Scarpati L. Winter J. Wuppermann A. (2016). *The Housing Crisis of the Late 2000s and Causal Paths Between Health and Socioeconomic Status.* Munich: University of Munich.

[B18] Ju D. Qin X. Xu M. Direnzo M. S. (2016). Boundary conditions of the emotional exhaustion-unsafe behavior link: the dark side of group norms and personal control. *Asia Pac. J. Manag.* 33 113–140. 10.1007/s10490-015-9455-7

[B19] Kahneman D. Tversky A. (1979). Prospect theory: an analysis of decision under risk. *Econometrica* 47 263–292. 10.2307/1914185

[B20] Kessler R. C. Brandenburg N. Lane M. Roy-Byrne P. Stang P. D. Stein D. J. (2005). Rethinking the duration requirement for generalized anxiety disorder: evidence from the national comorbidity survey replication. *Psychol. Med.* 35 1073–1082. 10.1017/S0033291705004538 16045073

[B21] Kessler R. C. Keller M. B. Wittchen H.-U. (2001). The epidemiology of generalized anxiety disorder. *Psychiatr. Clin. North Am.* 24 19–39. 10.1016/S0193-953X(05)70204-5 11225507

[B22] Lee J. J. Gino F. Staats B. R. (2014). Rainmakers: why bad weather means good productivity. *J. Appl. Psychol.* 99 504–513. 10.1037/a0035559 24417552

[B23] Leon A. C. Olfson M. Broadhead W. E. Barrett J. E. Blacklow R. S. Keller M. B. (1995). Prevalence of mental disorders in primary care: implications for screening. *Arch. Fam. Med.* 4 857–861. 10.1001/archfami.4.10.857 7551133

[B24] Lin C.-L. Chen C.-S. Liu T.-C. (2015). Do stock prices drive people crazy? *Health Policy Plan.* 30 206–214. 10.1093/heapol/czu007 24526705

[B25] Long B. C. Stavel R. V. (1995). Effects of exercise training on anxiety: a meta-analysis. *J. Appl. Sport Psychol.* 7 167–189. 10.1080/10413209508406963

[B26] Maslow A. H. (1943). A theory of human motivation. *Psychol. Rev.* 50 370–396. 10.1037/h0054346

[B27] McAfee A. Brynjolfsson E. (2012). Big data: the management revolution. *Harv. Bus. Rev.* 90 60–68. 23074865

[B28] McInerney M. Mellor J. M. Nicholas L. H. (2013). Recession depression: mental health effects of the 2008 stock market crash. *J. Health Econ.* 32 1090–1104. 10.1016/j.jhealeco.2013.09.002 24113241 PMC3874451

[B29] Olfson M. Fireman B. Weissman M. M. Leon A. C. Sheehan D. V. Kathol R. G. (1997). Mental disorders and disability among patients in a primary care group practice. *Am. J. Psychiatry* 154 1734–1740. 10.1176/ajp.154.12.1734 9396954

[B30] Petruzzello S. J. Landers D. M. Hatfield B. D. Kubitz K. A. Salazar W. (1991). A meta-analysis on the anxiety-reducing effects of acute and chronic exercise. *Sports Med.* 11 143–182. 10.2165/00007256-199111030-00002 1828608

[B31] Qin X. Huang M. Johnson R. E. Hu Q. Ju D. (2018). The short-lived benefits of abusive supervisory behavior for actors: an investigation of recovery and work engagement. *Acad. Manag. J.* 61 1951–1975. 10.5465/amj.2016.1325

[B32] Raedeke T. D. (2007). The relationship between enjoyment and affective responses to exercise. *J. Appl. Sport Psychol.* 19 105–115. 10.1080/10413200601113638

[B33] Roy-Byrne P. P. Wagner A. (2004). Primary care perspectives on generalized anxiety disorder. *J. Clin. Psychiatry* 65 20–26. 15384933

[B34] San J. (2006). Why Chinese people don’t want to seek psychiatric treatment? [in Chinese]. *Encyclopedic Knowl.* 10 7–8.

[B35] Schwandt H. (2014). Wealth shocks and health outcomes: evidence from stock market fluctuations. *Am. Econ. J. Appl. Econ.* 10 349–377. 10.1257/app.20140499

[B36] Spitzer R. L. Kroenke K. Williams J. B. W. Löwe B. (2006). A brief measure for assessing generalized anxiety disorder: the GAD-7. *Arch. Intern. Med.* 166 1092–1097. 10.1001/archinte.166.10.1092 16717171

[B37] Stevenson B. Wolfers J. (2013). Subjective well-being and income: is there any evidence of satiation? *Am. Econ. Rev.* 103 598–604. 10.1037/a0029873 23231724

[B38] Tversky A. Kahneman D. (1991). Loss aversion in riskless choice: a reference-dependent model. *Q. J. Econ.* 106 1039–1061. 10.2307/2937956

[B39] Wittchen H.-U. Zhao S. Kessler R. C. Eaton W. W. (1994). DSM-III—R generalized anxiety disorder in the national comorbidity survey. *Arch. Gen. Psychiatry* 51 355–364. 10.1001/archpsyc.1994.03950050015002 8179459

[B40] Wooldrige J. M. (2002). *Econometric Analysis of Cross Section and Panel Data.* Cambridge, MA: MIT Press.

[B41] www.cctv.com. (2007). *Paying Attention to Stock Market Investors: Buying Stocks is Like Playing the Heartbeat [in Chinese].* Available at: http://www.cctv.com/program/qqzxb/20070523/101321.shtml

[B42] Zayfert C. Dums A. R. Ferguson R. J. Hegel M. T. (2002). Health functioning impairments associated with posttraumatic stress disorder, anxiety disorders, and depression. *J. Nerv. Ment. Dis.* 190 233–240. 10.1097/00005053-200204000-00004 11960084

