# Supplementary material for: Stock Market Exposure and Anxiety in a Turbulent Market: Evidence From China
Source: Front Psychol. 2019 Feb 19;10:328. doi: 10.3389/fpsyg.2019.00328 (PMC6401606; doi:10.3389/fpsyg.2019.00328)
Supplement: Supplementary file 1 [file Table_1.docx]

**Supplemental Material**

Table S1. Means, Standard Deviations, and Correlations for Variables

| Variable | *M* | *SD* | Min | Max | 1 | 2 | 3 | 4 |
| --- | --- | --- | --- | --- | --- | --- | --- | --- |
| 1. SSE Composite Index (log) | 7.910 | 0.296 | 7.603 | 8.542 |  |  |  |  |
| 2. Amplitude of SSE Composite Index | 2.000 | 1.431 | 0.589 | 8.355 | 0.666^***^ |  |  |  |
| 3. Change amount of SSE Composite Index | 0.166 | 0.798 | –2.782 | 1.844 | 0.039 | –0.239^***^ |  |  |
| 4. Exposure to stock | 0.150 | 0.157 | 0.021 | 1.013 | 0.061^**^ | 0.047^*^ | –0.002 |  |
| 5. Anxiety disorder | 0.678 | 0.969 | 0.000 | 6.724 | 0.556^***^ | 0.468^***^ | –0.015 | 0.443^***^ |

*n* = 2542, from January 2014 to July 2015, total 82 weeks, for all models except exposure to stock; *n* = 2387, from January 2014 to June 2015, total 77 weeks, for models with exposure to stock. *t* statistics in parentheses. ^*^ significant at 5%, ^**^ significant at 1%, ^***^ significant at 0.1%.

Table S2. Impact of Exposure to Stock on Anxiety Disorder (Using the Keyword “Anxiety Disorder”) (Panel Analyses)

| Variable | Model 1 | Model 2 | Model 3 | Model 4 | Model 5 | Model 6 |
| --- | --- | --- | --- | --- | --- | --- |
| SSE Composite Index (log) | 0.565^***^ (6.967) |  |  | 0.587^***^ (5.672) |  | 0.326^*^ (2.243) |
| Amplitude of SSE Composite Index |  | 0.065^***^ (6.563) |  | –0.009 (–0.760) |  | –0.051^*^ (–2.141) |
| Change amount of SSE Composite Index |  |  | 0.072^**^ (3.595) | 0.057^**^ (2.906) |  | 0.075^**^ (3.366) |
| Exposure to stock |  |  |  |  | 13.614^***^ (4.895) | 11.480^**^ (3.524) |
| Constant | 1.570^*^ (2.449) | 5.908^***^ (301.492) | 6.024^***^ (1693.186) | 1.407^+^ (1.757) | 3.993^***^ (9.583) | 1.817^*^ (2.272) |
| *R*^2^ | 0.025 | 0.008 | 0.003 | 0.027 | 0.040 | 0.047 |
| *F* | 48.544^***^ | 43.071^***^ | 12.922^***^ | 18.193^***^ | 23.959^***^ | 22.439^***^ |

*n* = 2542, from January 2014 to July 2015, total 82 weeks, for all models except exposure to stock; *n* = 2387, from January 2014 to June 2015, total 77 weeks, for models with exposure to stock. *t* statistics in parentheses. ^*^ significant at 5%, ^**^ significant at 1%, ^***^ significant at 0.1%.

Table S3. Means, Standard Deviations, and Correlations for Variables in a Bull Market

| Variable | *M* | *SD* | Min | Max | 1 | 2 | 3 | 4 |
| --- | --- | --- | --- | --- | --- | --- | --- | --- |
| 1. SSE Composite Index (log) | 7.872 | 0.280 | 7.603 | 8.542 |  |  |  |  |
| 2. Amplitude of SSE Composite Index | 1.684 | 0.882 | 0.589 | 4.597 | 0.703^***^ |  |  |  |
| 3. Change amount of SSE Composite Index | 0.271 | 0.600 | –1.057 | 1.844 | 0.375^***^ | 0.248^***^ |  |  |
| 4. Exposure to stock | 0.149 | 0.156 | 0.021 | 1.013 | 0.054^**^ | 0.035 | 0.020 |  |
| 5. Anxiety disorder | 0.579 | 0.863 | 0.000 | 6.087 | 0.552^***^ | 0.416^***^ | 0.165^***^ | 0.433^***^ |

*n* = 2325, from January 1, 2014 to June 12, 2015; total 75 weeks. ^*^ significant at 5%, ^**^ significant at 1%, ^***^ significant at 0.1%.

Table S4. Impact of Exposure to Stock on Anxiety Disorder in a Bull Market (Panel Analyses)

| Variable | Model 1 | Model 2 | Model 3 | Model 4 | Model 5 | Model 6 |
| --- | --- | --- | --- | --- | --- | --- |
| SSE Composite Index (log) | 1.704^***^ (7.454) |  |  | 1.646^***^ (7.562) |  | 1.217^***^ (7.962) |
| Amplitude of SSE Composite Index |  | 0.407^***^ (7.344) |  | 0.051^**^ (3.212) |  | 0.064^***^ (3.784) |
| Change amount of SSE Composite Index |  |  | 0.237^***^ (6.244) | –0.069^***^ (–4.916) |  | –0.067^***^ (–4.871) |
| Exposure to stock |  |  |  |  | 29.616^***^ (5.742) | 13.232^**^ (3.624) |
| Constant | –12.837^***^ (–7.132) | –0.105 (–1.129) | 0.515^***^ (50.077) | –12.447^***^ (–7.229) | –3.832^***^ (–4.988) | –11.060^***^ (–9.193) |
| *R*^2^ | 0.407 | 0.231 | 0.036 | 0.411 | 0.294 | 0.448 |
| *F* | 55.559^***^ | 53.942^***^ | 38.987^***^ | 21.214^***^ | 32.966^***^ | 25.554^***^ |

*n* = 2325, from January 1, 2014 to June 12, 2015; total 75 weeks. *t* statistics in parentheses. ^*^ significant at 5%, ^**^ significant at 1%, ^** *^significant at 0.1%.

*Figure S1.* The amplitude of SSE Composite Index from January 2014 to July 2015.

*Figure S2.* The change amount for SSE Composite Index from January 2014 to July 2015.

*Figure S3.* The average number of accounts for all listed stocks (A shares) in the SSE and the SZSE from January 2014 to June 2015 in 31 provinces.

*Figure S4.* The change in the number of accounts for all listed stocks (A shares) in the SSE and the SZSE from January 2014 to June 2015.

*Figure S5.* The interaction of exposure to stock and weekly exercise frequency in predicting anxiety disorder in Study 2b.
